# Molecular phylogenetic analysis of the genus *Gloydius* (Squamata, Viperidae, Crotalinae), with description of two new alpine species from Qinghai-Tibet Plateau, China

**DOI:** 10.3897/zookeys.1061.70420

**Published:** 2021-10-04

**Authors:** Jing-Song Shi, Jin-Cheng Liu, Rohit Giri, John Benjamin Owens, Vishal Santra, Sourish Kuttalam, Melvin Selvan, Ke-Ji Guo, Anita Malhotra

**Affiliations:** 1 Key Laboratory of Vertebrate Evolution and Human Origins of Chinese Academy of Sciences, Institute of Vertebrate Paleontology and Paleoanthropology, Chinese Academy of Sciences, Beijing 100044, China; 2 Institute of Herpetology, Shenyang Normal University, Shenyang 110034, China; 3 Department of Zoology, Prithvi Narayan Campus, Bhimkalipatan-1, Pokhara 33700, Nepal; 4 Molecular Ecology and Evolution at Bangor, School of Natural Sciences, Bangor University, Bangor, Gwynedd LL57 2UW, UK; 5 Captive and Field Herpetology, Wales, UK; 6 Society for Nature Conservation, Research and Community Engagement (CONCERN), Nalikul, Hooghly, West Bengal, India; 7 Endangered Wildlife Trust, Dindigull, Tamil Nadu, India; 8 Central South Inventory and Planning Institute of National Forestry and Grassland Administration, College of Life Sciences and Technology, Central South University of Forestry and Technology, Changsha, Hunan 410004, China

**Keywords:** Asian pit viper, *Gloydiushimalayanus*, Heishui, molecular phylogeny, osteology, Qinghai-Tibet plateau, Zayu

## Abstract

We provide a molecular phylogeny of Asian pit vipers (the genus *Gloydius*) based on four mitochondrial genes (12S, 16S, ND4, and cytb). Sequences of *Gloydiushimalayanus*, the only member of the genus that occurs south of the Himalayan range, are included for the first time. In addition, two new species of the genus *Gloydius* are described based on specimens collected from Zayu, Tibet, west of the Nujiang River and Heishui, Sichuan, east of the Qinghai-Tibet Plateau. The new species, *Gloydiuslipipengi* sp. nov., can be differentiated from its congeners by the combination of the following characters: the third supralabial not reaching the orbit (separated from it by a suborbital scale); wide, black-bordered greyish postorbital stripe extending from the posterior margin of the orbit (not separated by the postoculars, covering most of the anterior temporal scale) to the ventral surface of the neck; irregular black annular crossbands on the mid-body; 23-21-15 dorsal scales; 165 ventral scales, and 46 subcaudal scales. *Gloydiusswild***sp. nov.** can be differentiated from its congeners by the narrower postorbital stripe (only half the width of the anterior temporal scale, the lower edge is approximately straight and bordered with white); a pair of arched stripes on the occiput; lateral body lakes black spots; a pair of round spots on the parietal scales; 21 rows of mid-body dorsal scales; zigzag dark brown stripes on the dorsum; 168–170 ventral scales, and 43–46 subcaudal scales. The molecular phylogeny in this study supports the sister relationship between *G.lipipengi***sp. nov.** and *G.rubromaculatus*, another recently described species from the Qinghai-Tibet Plateau, more than 500 km away, and indicate the basal position of *G.himalayanus* within the genus and relatively distant relationship to its congeners.

## Introduction

Asian pit vipers of the genus *Gloydius* Hoge & Romano Hoge, 1978 are small-bodied venomous snakes distributed mainly in northern Asia, but extending into southern Europe in the case of *G.halys*. They are quite common and have radiated into various habitats. At present, more than 20 species mainly belonging to three species groups (i.e., the *G.blomhoffii* complex, *G.intermedius–halys* complex, and *G.strauchi* complex) are recognized (Orlov and Barabanov 1999; Zhao 2006; Shi et al. 2017, 2018). Within *Gloydius*, most species having 21 rows of mid-body dorsal scales and three palatine teeth have been attributed to many subspecies of *G.strauchi* (Bedriaga, 1912). Recently, several former subspecies have been elevated to full species (e.g., *G.qinlingensis* (Song & Chen, 1985)), *G.liupanensis* (Liu, Song & Luo, 1989), and *G.monticola* (Werner, 1922)) and several new species have been described from across the range of the complex (e.g., *G.rubromaculatus*, *G.angusticeps*, and *G.huangi*; Xu et al. 2012; Shi et al. 2017; Shi et al. 2018; Wang et al. 2019).

Given that the *Gloydiusstrauchi* complex is widely distributed in western China (Zhao et al.1998; Zhao 1999, 2006), some of the specimens from previous studies are now attributable to the recently elevated species described above. The distribution of *G.strauchi* sensu stricto has been restricted to western Sichuan by recent molecular and morphological studies (Orlov and Barabanov 1999, 2000; Shi et al. 2017, 2018). With respect to Tibet, older records of the *G.strauchi* complex refer to at least two different species, *G.rubromaculatus* from Jiangda (Shi et al. 2017, 2018) and *G.huangi* from Chamdo (Wang et al. 2019). However, given this wide-ranging complex spans several biogeographic barriers and distinct environments in a poorly investigated region, we hypothesize that there still might be hidden species within the *G.strauchi* complex.

Additionally, *Gloydiushimalayanus* (Günther, 1864) has long been regarded as a full species within the *G.strauchi* complex based on its unique morphological characters (e.g., the conspicuous rostralis and the triangular head in dorsal view; Gloyd and Conant 1990). In spite of numerous recent studies focused on the molecular phylogeny of the genus *Gloydius* (Xu et al. 2012; Eskandar et al. 2018; Shi et al. 2016, 2018; Asadi et al. 2019; Wang et al. 2019), the systematic and taxonomic position of *G.himalayanus* in relation to the *G.strauchi* complex is still unclear due to lack of the sequence data for this species.

In this study, we use a molecular phylogeny of *Gloydius*, including data of *G.himalayanus* for the first time, and provide a description of two new species from the *Gloydiusstrauchi* complex from Zayu, Tibet, and Heishui, Sichuan, China.

## Materials and methods

### Specimen collection

We examined preserved specimens from the Chengdu Institute of Biology (**CIB**), Kunming Institute of Zoology (**KIZ**), Institute of Zoology (**IOZ**), and Shenyang Normal University (**SYNU**). Newly obtained specimens were deposited in the Institute of Vertebrate Paleontology and Paleoanthropology (**IVPP**), Beijing.

### Morphology

Snout–vent length (SVL), tail length (TL), and total length (TTL; i.e., SVL + TL) were measured with a flexible ruler to the nearest 1 mm. Other morphological measurements were taken with 0–200 mm vernier calipers to the nearest 0.1 mm: head length (HL, from the tip of snout to the posterior margin of mandible), head width (HW, the widest part of the head in dorsal view), head depth (HD, the deepest part of the head in lateral view), snout length (SL, from the tip of snout to the anterior margin of the eye), eye diameter (ED, measured as a horizontal distance), interorbital space (IOS, the distance between the top margin of eyes), and internasal space (INS, the distance between nostrils). Numbers of supralabials (SPL), infralabials (IFL), dorsal scales (DS), ventral scales (V, excluding four preventral scales), and subcaudal scales (SC) were counted.

### X-ray scanning and three-dimensional reconstructions

The scanning was carried out with 225-kV micro-computerized tomography, developed by the Institute of High Energy Physics (IHEP), CAS. A total of 720 transmission images were reconstructed into the 2048 × 2048 matrix of 1536 slices using two-dimensional reconstruction software developed by the IHEP, CAS. The final CT reconstructed skull model was exported with a minimum resolution of 26.7 μm.

### DNA extraction, polymerase chain reaction (PCR) and sequencing

Tissue samples for molecular analyses were taken separately and preserved in 95% ethanol at −40 °C. Genomic DNA was extracted with Qiaprep Spin Miniprep kit (QiaGen). Four mitochondrial genome fragments were specifically amplified for this study: a 859 bp fragment of 12S ribosomal RNA (12S), using primers 12SFPhe and 12SRVal, described by Knight and Mindell (1993); a 465 bp fragment of 16S ribosomal RNA (16S) using primers 16sFL and 16sRH described by Palumbi et al. (1991); a 1065 bp fragment of cytochrome *b* (cytb) using primers L14919 and H16064 described by Burbrink et al. (2000), and a 666 bp fragment of NADH dehydrogenase subunit 4 (ND4), using the primers ND4 and Leu, described by Arevalo et al. (1994). The standard PCR protocol was performed in a 20 µl reaction with at least 20 ng of template DNA and 10 pmol of primers. PCR conditions consisted of an initial denaturation for 3 min at 94 °C, followed by 35 cycles of denaturation at 94 °C for 30 sec, 30 sec of annealing at primer-specific temperatures (56 °C for ND4, 54 °C for 16S, 48 °C for cytb), and extension at 72 °C for 60 sec, finalized with an extension step of 10 min at 72 °C. Sequencing was conducted by Beijing Tianyi Huiyuan Bio-tech Co., Ltd.

### Phylogenetic analyses

We use 46 individuals of the 22 recognized *Gloydius* species, except for unavailable sequence data of *G.halysboehmei* (Nilson, 1983), in a phylogenetic. In order to establish the monophyly of *Gloydius*, 12 additional species of outgroups from the family Viperidae (i.e., *Calloselasma*, *Deinagkistrodon*, *Ovophis*, *Protobothrops*, *Sinovipera*, *Trimeresurus*, *Viridovipera*, and *Vipera*) were also included.

Sequence data obtained from GenBank and from this study are listed in Table 2. Sequences were aligned in MEGA6 (Tamura et al. 2013). With respect to the different evolutionary characters of each molecular marker, the dataset was initially split into eight partitions by gene and codon positions, and then combined into five partitions taking advantage of PartitionFinder 2.1.1 (Lanfear et al. 2012) to find similarly evolving partitions.

Bayesian phylogenetic analysis was performed using MrBayes 3.1.2 (Ronquist et al. 2011). All searches consist of three heated chains and a single cold chain. Three independent iterations each comprising two runs of 100,000,000 generations were performed, sampling every 10,000 generations, and parameter estimates were plotted against generation. The first 25% of the samples were discarded as burn-in, resulting in a potential scale reduction factor (PSRF) of <0.005. Maximum likelihood analysis was run with the IQtree tool in the web server CIPRES (https://www.phylo.org/index.php), with 1,000 fast bootstrap repeats.

General time reversible (GTR) model, the most probable substitution model for the corrected ND4*p*-distance matrix was calculated in PAUP 4.0.

## Results

### Morphology

**Figure 1.**
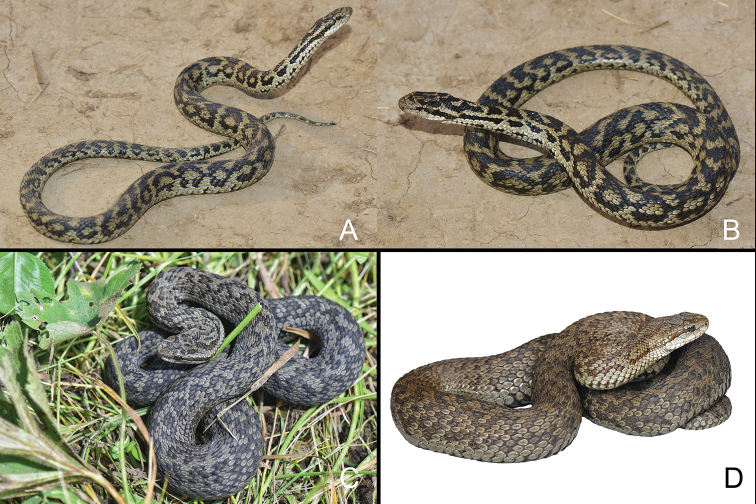
*Gloydiuslipipengi* sp. nov. (**A, B**IVPP OV 2720, holotype) and *Gloydiusswild* sp. nov. (**C**IVPP OV 2725, holotype, **D**IVPP OV, 2726, paratype) in life, not to scale.

**Figure 2.**
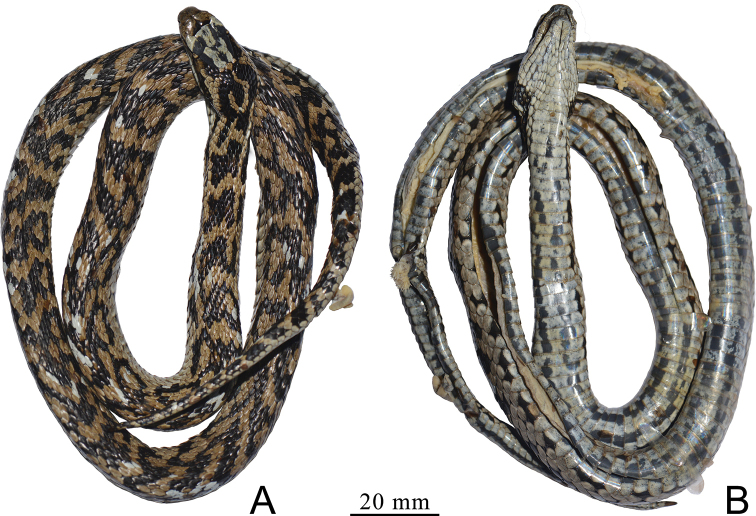
Holotype of *Gloydiuslipipengi* sp. nov. (IVPP OV 2720) in preservative **A** dorsal view **B** ventral view.

**Figure 3.**
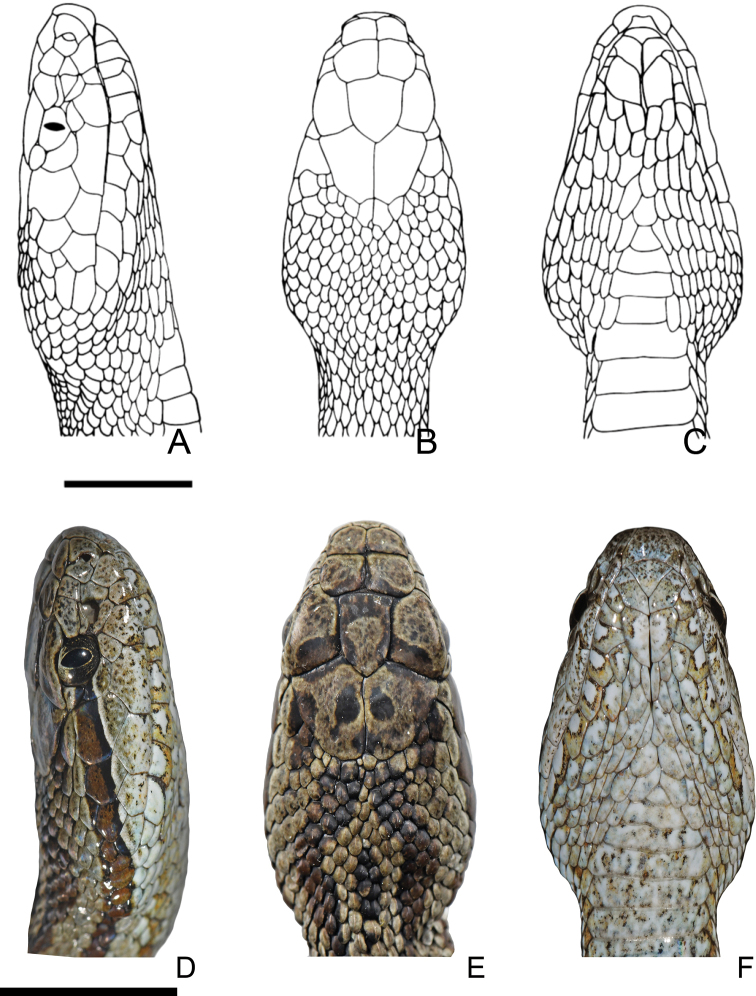
Head squamation of *Gloydiuslipipengi* sp. nov. (Holotype, IVPP OV 2720: **A** lateral view **B** dorsal view **C** ventral view) and *G.swild* sp. nov. (Holotype, IVPP OV 2725: **D** lateral view **E** dorsal view **F** ventral view). Scale bar: 10 mm.

**Figure 4.**
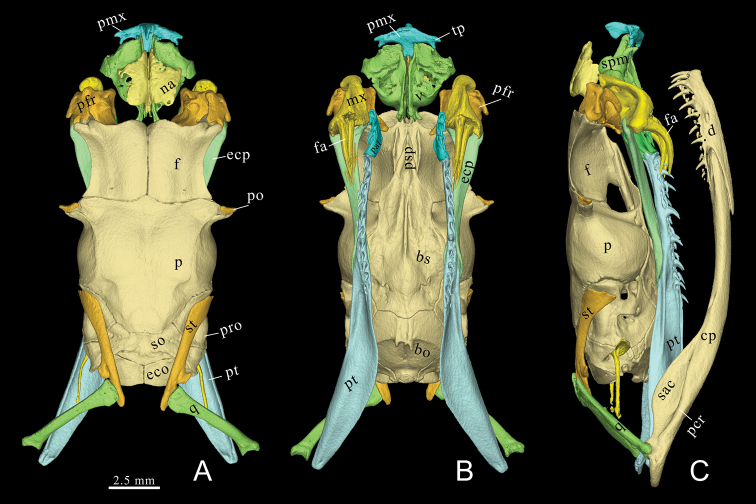
Color rendered three-dimensional model of *Gloydiuslipipengi* sp. nov. (holotype, IVPP OV 2720) **A** dorsal view **B** palatal view, mandibles not shown **C** lateral view. Abbreviations: bo, basioccipital; bs, basisphenoid; col, columella; cp, compound bone; d, dentary; ecp, ectopterygoid; exo, exoccipital; f, frontal; na, nasal; ma, maxilla; p, parietal; pcr, prearticular crest of compound bone; pfr, prefrontal; pmx, premaxilla; po, postorbital; pp, palatine process of maxilla; pro, prootic; psp, parasphenoid rostrum; pt, pterygoid; sac, surangular crest of compound bone; spm; septomaxilla; so, supraoccipital; sp, splenial; st, supratemporal; v, vomer. Conducted by Ye-Mao Hou and Jingsong Shi.

Comparative data of specimens examined are listed in Table 1 and the holotypes are illustrated in Figures 1–4.

**Table 1.** Comparison of specimens of the *Gloydiusstrauchi* complex.

Taxa	Museum vouchers	Preserve	Localities	Sex	SVL	TTL	TL	HL	HW	HH	SL	ED	IOS	INS	V	Sc	DS	SPL (L/R)	IFL (L/R)	Reference
*Gloydiuslipipengi* sp. nov.	IVPP OV 2720**	IVPP	Zawalong, Zayu, Tibet	M	540.6	628.2	87.6	25.2	13.2	8.2	7.4	2.9	9.6	5.4	165	46	23-21-15	7/7	10/11	This study
*G.swild* sp. nov.	IVPP OV 2725**	IVPP	Heishui, Aba, Sichuan	F	462.0	529.5	67.5	20.8	12.2	6.6	5.8	2.4	7.6	4.1	170	46	21-21-15	7/7	10/10	This study
	IVPP OV 2726*	IVPP	Heishui, Aba, Sichuan	F	552.0	629.1	77.1	23.8	15.7	8.4	6.2	3.2	9.6	5.0	168	43	21-21-17	7/7	10/10	This study
*G.angusticeps*	IVPP OV 2634**	IVPP	Xiaman, Sichuan	M	373.2	439.7	66.5	21.2	12.4	6.6	6.7	2.2	9.1	4.1	148	39	19-19-15	7/7	10/10	Shi et al. (2018)
	JS1507G5A*	SYNU	Xiaman	M	283.4	331.6	42.2	16.9	9.8	6.3	4.5	2.0	7.5	3.3	151	39	19-20-15	6/6	9/10	Shi et al. (2018)
	JS1306G1A*	SYNU	Golog, Qinghai	F	443.1	502.3	59.2	23.6	13.2	7.0	5.3	2.8	8.3	4.3	162	31	21-21-15	7/6	8/9	Shi et al. (2018)
	IOZ002317*	IOZ	Golog, Qinghai	F	457.2	459.4	72.2	22.1	11.8	7.1	—	—	8.0	4.5	157	35	19-21-15	6/6	10/10	Shi et al. (2018)
*G.huangi*	KIZ 027654**	KIZ	Chaya, Chamdo, Tibet,	F	532.0	455.0	67.0	23.2	14.6	—	—	3.1	8.4	4.3	174	43	21-21-15	7/7	10/10	Wang et al. (2019)
*G.monticola*	CIB72553	CIB	Zhongdian, Yunnan	F	274.0	308.0	34.0	18.1	9.5	6.4	—	1.5	6.9	4.7	145	30	21-21-15	6/6	9/10	Shi et al. (2017)
*G.rubromaculatus*	IOZ 032317**	IOZ	Yushu, Qinghai	M	473.0	554.0	81.0	24.6	15.8	7.4	7.8	3.1	8.2	4.6	158	43	21-21-15	7/8	10/11	Shi et al. (2017)
*G.strauchi*	SUNU1410G3△	SYNU	Kangding, Sichuan	M	407.3	482.7	75.4	21.5	13.4	7.8	—	2.8	9.3	4.4	144	45	21-21-15	7/7	10/10	Shi et al. (2017)
	CIB14356△	CIB	Kangding	M	338.5	405.0	66.3	19.4	11.8	6.2	—	2.1	7.7	4.2	151	38	21-21-16	7/7	—	Shi et al. (2017)
	CIB14357△	CIB	Kangding	M	347.2	412.4	65.2	19.9	12.1	8.7	—	2.2	7.8	3.7	146	41	21-21-15	7/7	—	Shi et al. (2017)
	SYNU1508G4	SYNU	Litang, Sichuan	M	372.3	436.4	64.1	20.3	12.7	6.5	5.9	2.1	8	4.3	148	42	21-21-15	7/7	10/10	Shi et al. (2017)
	CIB78588	CIB	Litang, Sichuan	M	427.3	504.6	77.3	24.6	15.6	8.2	—	2.7	9.9	5.3	151	40	21-21-16	7/7	10/10	Shi et al. (2017)
	CIB14358△	CIB	Kangding, Sichuan	F	384.1	438.3	54.2	22.4	12.4	7.9	—	2.4	8.4	5.6	158	35	21-21-15	7/7	—	Shi et al. (2017)
	CIB14359△	CIB	Kangding, Sichuan	F	450.3	505.5	55.2	20.9	12.4	7.2	—	1.9	7.8	6	160	33	21-21-15	7/7	—	Shi et al. (2017)

Note: **, holotype; *, paratype; △, topotype. Dimensions are measured to the nearest 0.1 mm.

### Molecular phylogeny

**Figure 5.**
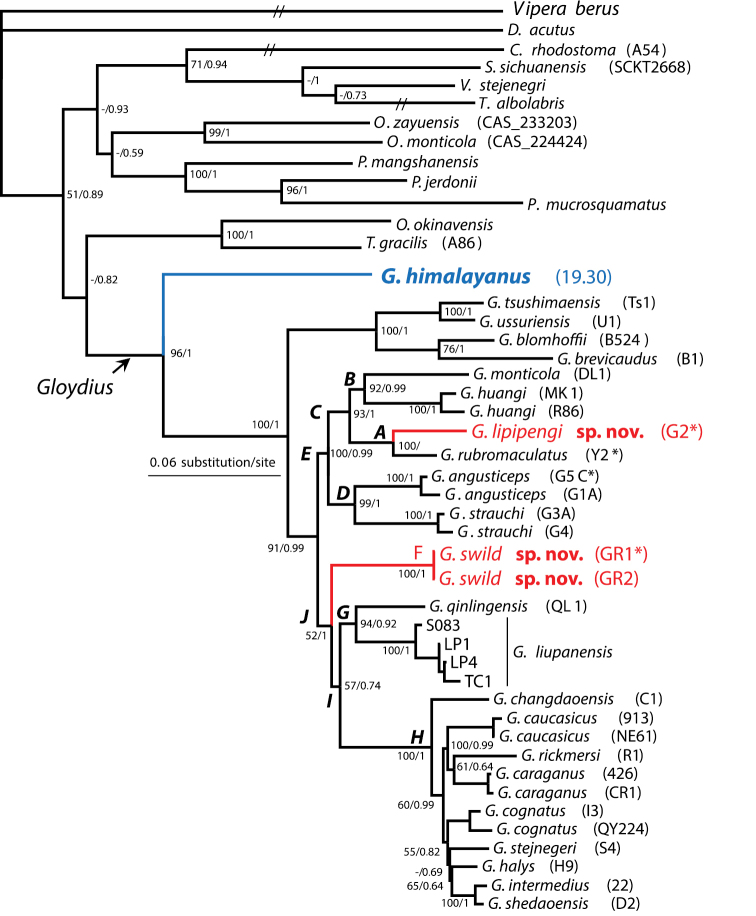
Bayesian inferenced tree of the genus *Gloydius*, along with some relative genus of the family Viperidae, based on 12S, 16S, ND4, and cytb sequences, with the maximum likelihood bootstrap supports (left, regular) and Bayesian posterior probabilities (right, italic) displayed on the nodes (those <50% are displayed as “-”). Holotypes are marked with asterisks.

Novel sequences were uploaded to GenBank and are available under accession numbers shown in Table 2, along with accession numbers for data obtained from GenBank. The final molecular dataset consisted of 3,065 bases containing 46 specimens. The evolutionary models assigned to each of the five partitions by PartitionFinder are shown in Table 3. The phylogeny from the Bayesian analysis (BI, Fig. 5) matches those given in earlier studies of the genus (Xu et al. 2012; Shi et al. 2017, 2018; Wang et al. 2019), except for the systematic position of the *G.qinlingensis-liupanensis* group, which do not form a monophyletic group with other members of the *G.strauchi* complex.

**Table 2.** Details of the molecular samples used in this study.

Taxa	Museum voucher	Code	Locality	locus				Reference
				*12s*	*16s*	cytb	ND4	
*Gloydiuslipipengi* sp. nov.	IVPP OV 2720**	G2	Zawalong, Zayu, Tibet	KY040542	KY040574	KY040628	KY040649	This study
*G.swild* sp. nov.	IVPP OV 2725**	GR1	Heishui, Aba, Sichuan	OK210582	OK184551	OK239647	OK239652	This study
	IVPP OV 2726*	GR2	Heishui, Aba, Sichuan	OK210583	OK184552	OK239648	OK239653	This study
*G.angusticeps*	JS1306G1A*	G1A	Golog, Qinghai	KY040541	KY040572	KY040627	KY040647	Shi et al. (2018)
	IVPP OV 2634**	G5C	Zoige, Sichuan	KY040545	KY040577	KY040631	KY040652	Shi et al. (2018)
*G.blomhoffii*	B524	B524	Japan	AY352719	AY352719	AY352751	AY352814	Malhotra (2003)
*G.brevicaudus*	CIB-DL70	B1	Liaoning	KY040552	KY040584	HQ528467	HQ528303	Shi et al. (2017)
*G.caraganus*	CR1	CR1	Kazakhstan	—	—	MF490455	MF490453	Shi et al. (2017)
	RIZ20426.1	426	Kyzylorda, Kazakhstan	MZ958021	MZ957012	MZ959165	MZ959158	This study
	RIZ29913	913	Mazandaran, Iran	MZ958022	MZ957013	MZ959166	MZ959159	This study
	NEZMUT_61	NE61	Alborz, Iran	—	—	MH378692	MH378729	Asadi et al. (2019)
*G.changdaoensis*	SYNUSHF01△	C1	Changdao, Shandong	KY040522	KY040554	KX063823	KX063796	Shi et al. (2017)
*G.cognatus*	CIB-QY224	QY224	Zoige, Sichuan	KY040529	KY040561	KY040619	KY040640	Shi et al. (2017)
	SYNU13109I3	I3	Saihan, Inner Mongolia	KY040531	KY040563	KY040621	KY040642	Shi et al. (2017)
*G.shedaoensis*	SYNU110D2△	D2	Lvshun, Liaoning	KY040523	KY040555	KX063819	KX063792	Shi et al. (2017)
*G.halyshalys*	SYNU 1510151	H9	Greater Xing’an, Heilongjiang	KY040528	KY040560	KY040618	KY040639	Shi et al. (2017)
*G.himalayanus*	—	19.30	Himachal Pradesh, India	MZ958982	MZ958980	MZ959172	MZ959173	This study
*G.huangi*	R84	R84	Mangkang, Tibet	—	MZ957017	MW732035	MZ355578	This study
	KIZ 027654*	027654	Chaya, Chamdo, Tibet	MK227409	MK227412	MK227415	MK227418	Wang et al. (2019)
*G.intermedius*	SYNU150622**	22	Zhuanghe, Liaoning	KY040524	KY040556	KY040617	KY040638	Shi et al. (2017)
*G.liupanensis*	S083	S083	Ningxia	—	MK193903	MK201255	JQ687472	Xu et al. (2012)
	LP1	LP1	Guyuan, Gansu	MZ958024	MZ957015	MZ959168	MZ959161	This study
	LP4	LP4	Guyuan, Gansu	MZ958025	MZ957016	MZ959169	MZ959162	This study
	TC1	TC1	Tanchang, Gansu	MZ958023	MZ9570124	MZ959167	MZ959160	This study
*G.monticola*	SYNU1607DL1	DL1	Dali, Yunnan	KY040549	KY040581	KY040635	MG025935	Shi et al. (2017)
*G.qinlingensis*	SYNUQL1△	QLS	Xunyangba, Shanxi	KY040534	KY040566	KY040623	KY040644	Shi et al. (2017)
*G.rickmersi*	MHNG 2752.69	R1	Kyrgyzstan	—	—	—	KM078592	Wagner et al. (2015)
*G.rubromaculatus*	IOZ032317**	Y2	Qumarleb, Qinghai	KY040546	KY040578	KY040632	KY040653	Shi et al. (2017)
*G.stejnegeri*	SYNU1508S4△	S4	Linfen, Shanxi	KY040537	KY040569	KX063818	KX063791	Shi et al. (2017)
*G.strauchi*	SYNU1501G3△	G3	Kangting, Sichuan	KY040543	KY040575	KY040629	KY040650	Shi et al. (2017)
*G.strauchi*	SYNU1508G4	G4	Litang, Sichuan	KY040544	KY040576	KY040630	KY040651	Shi et al. (2017)
*G.tsushimaensis*	—	Ts1	Japan	JN870203	JN870196	JN870203	JN870211	Fenwick (2011)
*G.ussuriensis*	U1	U1	Heilongjiang	KP262412	KP262412	KP262412	KP262412	Xu et al. (2012)
*Calloselasmarhodostoma*			unknown	AY352779	AY352718	AY352813	—	Directly submitted
*Deinagkistrodonacutus*	—	A	Fujian	DQ343647	DQ343647	DQ343647	DQ343647	Yan et al. (2008)
*Ovophismonticola*	CAS_224424	—	Yunnan, China	HQ325303	HQ325117	HQ325238	HQ325176	Malhotra et al. 2011
*O.zayuensis*	CAS_233203	—	Tibet, China	HQ325304	HQ325118	HQ325239	HQ325177	Directly submitted
*Ovophisokinavensis*	—	—	Japan	AB175670	AB175670	AB175670	AB175670	Directly submitted
*Protobothropsjerdonii*	—	—	Guangdong, China	NC021402	NC021402	NC021402	NC021402	Directly submitted
*P.mangshanensis*	—	—	Hunan, China	NC026052	NC026052	NC026052	NC026052	Directly submitted
*P.mucrosquamatus*	—	—	Guangdong, China	NC021412	NC021412	NC021412	NC021412	Directly submitted
*S.sichuanensis*	SCKT2668	SCKT2668	Sichuan	KT2668	KT2668	KT2668	KT2668	Zhu et al. (2015)
*Trimeresurusalbolabris*	—	—	Guangdong, China	NC022820	NC022820	NC022820	NC022820	Directly submitted
*T.gracilis*	—	A86	Taiwan, China	AY352789	AY352728	AY352823	—	Directly submitted
*Viridoviperastejenegri*	—	—	Taiwan, China	FJ752492	FJ752492	FJ752492	FJ752492	Directly submitted
*Viperaberus*	—	—	—	NC036956	NC036956	NC036956	NC036956	Directly submitted

Note: **, holotype; *, paratype; △, topotype. The data that not obtained are marked as “—”.

**Table 3.** Partitions and their evolutionary models selected by PartitionFinder 2.1.1.

Partitions	Locus	Length (bp)	Models
Partition 1	12S	1,435	GTR+I+G
Partition 2	16S	475	GTR+I+G
Partition 3	cytb pos1, ND4 pos1	577	TVM+I+G
Partition 4	cytb pos2 and ND4 pos2	577	TVM+I+G
Partition 5	ND4 pos3 and cytb pos3	577	TIM+G

GTR: General Time-Reversible model; TVM: transversional substitution model; TIM: transitional substitution model.

In this study, the topological structures of the maximum likelihood (ML) and Bayesian inference (BI) trees are generally consistent. The lineage of the new specimen from Zayu, Tibet (G2), constitutes a sister group to the clade of *G.rubromaculatus* from Sanjiangyuan, Qinghai (Y2), but is separated from it by significant branch lengths. The clade including *G.lipipengi* sp. nov. and *G.rubromaculatus* (Clade A) is sister to the clade formed by *G.huangi* and *G.monticola* (Clade B), forming a monophyletic lineage (Clade C). Clade C is sister to the monophyletic clade constituted by *G.strauchi* and *G.angusticeps* (Clade D), forming another monophyletic clade (Clade E).

The two new specimens from Heishui, Sichuan (GR1 and GR2), forming a strongly supported monophyletic group (Clade F). The clade of *G.qinlingensis* is sister to the clades of *G.liupanensis*, forming Clade G. The samples of the nine species of *G.halys-intermedius* group constitute another monophyletic group, Clade H. Clade G is sister to Clade F, forming a monophyletic Clade (Clade I) sister to the clade constituted by the new specimens from Heishui (Clade F), forming Clade J.

The phylogenetic position of *Gloydiushimalayanus*, the only species of the genus to be found on the southern slopes of the Himalayan ranges, is basal to, and considerably distant from other species of *Gloydius* (13–16.1% *p*-distance for ND4, Table 4), although the genus as a whole is well supported as a monophyletic group in this analysis.

The corrected *p*-distance between the new specimen from Zayu, Tibet and *G.rubromaculatus* sequences is greater than those between other recognised species (4.4% for ND4, Table 4); the corrected *p*-distances between the new specimens from Heishui and one of its closest related congeners, *G.rubromaculatus*, are greater than those between other recognised species (8.5% for ND4, Table 4). Thus, the molecular phylogeny supports these new specimens from both Zayu and Heishui as phylogenetically independent species.

**Table 4.** Corrected distance among *Gloydius* species (ND4, based on the general time-reversible [GTR] model). Values between *Gloydiuslipipengi* sp. nov., *G.swild* sp. nov. and their congeners are highlighted in bold type.

		1	2	3	4	5	6	7	8	9	10	11	12	13	14	15	16	17	18	19	20	21	22
1	*G.intermedius* (22)	-																					
2	*G.shedaoensis* (D2)	0.011	-																				
3	*G.halys* (H9)	0.041	0.042	-																			
4	*G.cognatus* (I3)	0.033	0.033	0.033	-																		
5	*G.stejnegeri* (S4)	0.045	0.050	0.047	0.041	-																	
6	*G.rickmersi* (R1)	0.052	0.051	0.054	0.049	0.065	-																
7	*G.caraganus* (CR1)	0.038	0.046	0.049	0.042	0.059	0.050	-															
8	*G.changdaoensis* (C1)	0.054	0.049	0.050	0.042	0.069	0.066	0.054	-														
9	*G.qinlingensis* (QL1)	0.110	0.122	0.106	0.104	0.113	0.113	0.114	0.113	-													
10	*G.liupanensis* (LP1)	0.090	0.099	0.088	0.086	0.097	0.102	0.097	0.095	0.039	-												
11	*G.strauchi* (G3A)	0.098	0.110	0.102	0.097	0.111	0.116	0.107	0.105	0.074	0.059	-											
12	*G.angusticeps* (G5C)	0.101	0.112	0.097	0.099	0.112	0.104	0.102	0.104	0.063	0.068	0.067	-										
13	*G.monticola* (DL1)	0.120	0.130	0.122	0.112	0.137	0.134	0.135	0.120	0.076	0.078	0.079	0.076	-									
14	*G.huangi* (R86)	0.111	0.123	0.117	0.112	0.122	0.122	0.119	0.124	0.081	0.080	0.086	0.080	0.078	-								
15	*G.rubromaculatus* (Y2)	0.106	0.115	0.100	0.109	0.118	0.109	0.113	0.112	0.086	0.085	0.090	0.079	0.089	0.085	-							
16	*G.lipipengi* sp. nov. (G2)	**0.112**	**0.125**	**0.114**	**0.127**	**0.124**	**0.121**	**0.119**	**0.132**	**0.078**	**0.081**	**0.092**	**0.081**	**0.088**	**0.089**	**0.044**	-						
17	*G.swild* sp. nov. (GR1)	**0.116**	**0.125**	**0.110**	**0.108**	**0.102**	**0.123**	**0.114**	**0.119**	**0.099**	**0.085**	**0.089**	**0.086**	**0.089**	**0.103**	**0.085**	**0.097**	-					
18	*G.swild* sp. nov. (GR2)	0.116	0.125	0.110	0.108	0.102	0.123	0.114	0.119	0.099	0.085	0.089	0.086	0.089	0.103	0.085	**0.097**	**0.000**	-				
19	*G.brevicaudus* (B1)	0.143	0.152	0.135	0.145	0.155	0.150	0.145	0.156	0.117	0.113	0.124	0.121	0.122	0.138	0.124	**0.126**	**0.138**	0.138	-			
20	*G.ussuriensis* (U1)	0.106	0.116	0.131	0.115	0.133	0.133	0.119	0.123	0.119	0.109	0.110	0.102	0.122	0.105	0.110	**0.128**	**0.118**	0.118	0.107	-		
21	*G.blomhoffii* (B524)	0.132	0.144	0.148	0.142	0.157	0.147	0.135	0.144	0.119	0.110	0.117	0.110	0.112	0.119	0.116	**0.125**	**0.125**	0.125	0.117	0.068	-	
22	*G.tsushimaensis* (Ts1)	0.121	0.135	0.145	0.133	0.152	0.142	0.139	0.146	0.126	0.113	0.118	0.108	0.110	0.121	0.130	**0.138**	**0.133**	0.133	0.122	0.054	0.053	-
23	*G.himalayanus* (19.30)	0.141	0.149	0.149	0.135	0.159	0.153	0.161	0.134	0.130	0.134	0.142	0.154	0.152	0.139	0.142	**0.146**	**0.146**	0.148	0.148	0.156	0.160	0.135

### Taxonomic account

**Viperidae Gray, 1825**

#### *Gloydius* Hoge & Romano-Hoge, 1981

##### Gloydius lipipengi

Taxon classificationAnimaliaSquamataViperidae

Shi, Liu & Malhotra sp. nov.

01E6E10A-ADF7-59CA-A589-BD6DFEDF3CAA

http://zoobank.org/6DF30D06-937B-470B-AFE4-D4CABEAF7DAB

###### Etymology.

The specific epithet of the new species from Tibet is dedicated to the senior author’s Master’s supervisor, Professor Pi-Peng Li (Institute of Herpetology, Shenyang Normal University) on Li’s sixtieth birthday. Prof. Li has devoted himself to the study of the herpetological diversity of the Qinghai-Tibet Plateau. The senior author became an Asian pit viper enthusiast and professional herpetological researcher under his instruction. The common name of *Gloydiuslipipengi* sp. nov. is suggested as “Nujiang pit viper” in English, and “Nù Jiāng Fù (怒江蝮)” in Chinese.

###### Type specimen.

*Gloydiuslipipengi* sp. nov., holotype. IVPP OV2720 (G2, Figs 1–4), adult male, collected from Muza Village, Zayu, Nyingchi Prefecture, Tibet (28.54°N, 98.23°E, 2883 m), by Jin-Cheng Liu, on 8 September 2014.

###### Diagnosis.

The specimens of the new species, IVPP OV 2720, IVPP OV 2725 and IVPP OV 2726 were identified as the member of the genus *Gloydius* based on the small body size, bilateral pits, and divided subcaudal scales (Hoge and Romano-Hoge 1981).

*Gloydiuslipipengi* sp. nov. differs from other congeneric species in the following characteristics: i) third supralabial scale not touching the orbit; ii) a pair of prominent black markings on the occiput; iii) black-bordered greyish cheek stripe extending from the posterior margin of orbit (not separated by the postoculars) to the ventral surface of the neck; iv) black irregular annular crossbands on the mid-body; iv) two rows of black blotches on the ventral side ; v) 23-21-15 circum-body scales; vi) 165 ventral scales; and vii) 46 subcaudal scales.

Table 5 provides a brief summary of the differences between *G.lipipengi* sp. nov., *G.swild* sp. nov. and other congeneric species.

**Table 5.** Brief morphological comparisons between *Gloydiuslipipengi* sp. nov., *G.swild* sp. nov. and other congeneric species.

Species	Dorsal head	Spots on supralabials	Canthus rostralis	Background coloration	Dorsal color patterns
*G.lipipengi* sp. nov.	triangular	greyish brown discrete spots	inconspicuous	greyish brown	irregular large black interlaced patches
*G.swild* sp. nov.	triangular	greyish brown discrete spots	inconspicuous	greyish brown or blueish-grey	irregular zigzag dark brown markings
*G.angusticeps*	triangular	greyish brown, vaporous spots	inconspicuous	greyish brown or yellowish brown	dark brown small blotches, large patcher or stripes
*G.himalayanus*	triangular	one triangular spot between the third and fourth supralabial	very conspicuous	reddish brown or light grey	brown, reddish brown or dark grey small blotches or large black bordered patches
*G.huangi*	rounded	yellowish brown small spots	inconspicuous	light or pale greyish yellow	regular large greyish brown patches
*G.liupanensis*	triangular	none	conspicuous	light reddish brown or yellowish brown	similar with qinlingensis, with white stripe on the body side
*G.monticola*	rounded	whitish borders of the labials along the mouth line	inconspicuous	greyish brown, greenish brown or reddish brown	dark grey or dark brown blotches or zigzag stripes
*G.qinlingensis*	triangular	none	conspicuous	light reddish brown or yellowish brown	two columns of irregular yellowish brown or dark brown
*G.rubromaculatus*	rounded	irregular small black spots	inconspicuous	khaki or yellowish brown	regular large (or discrete small) scarlet or brownish yellow patches or stripes
*G.strauchi*	rounded	large brown between the second, third and fourth or none	inconspicuous	greenish brown, yellowish brown or nut-brown	patches, transverse crossbands or four longitudinal zigzag strips

*Gloydiuslipipengi* sp. nov. and *G.swild* sp. nov. can be differentiated from the species in the *G.blomhoffii* complex by having three palatine teeth (versus four palatine teeth), from the *G.halys* complex by having 21 rows of mid-body dorsal scales (versus 22 or 23 rows). *Gloydiuslipipengi* sp. nov. differs from other species in *G.strauchi* complex by the third supralabial scale not touching the orbit, from *G.strauchi*, *G.huangi*, and *G.rubromaculatus* by having large irregular black markings on the back (versus four irregular longitudinal stripes or discrete blotches in *G.strauchi*, complete dark brown patches in *G.huangi*, and large red crossbands in *G.rubromaculatus* (Wang et al. 2019), from *G.monticola* by having seven supralabials (versus always six supralabials) and more subcaudal scales (46 pairs versus always fewer than 30 pairs), from *G.qinlingensis* and *G.liupanensis* by its greyish brown body colour (versus yellowish-brown body colour) and lacking a lateral white line on each lateral side (versus possessing a lateral white line on each side). *Gloydiuslipipengi* sp. nov. can be differentiated from *G.himalayanus* by possessing an indistinct canthus rostralis (versus very distinct canthus rostralis; Gloyd and Conant 1990).

###### Description of the holotype.

IVPP OV 2720, adult male, a slender pit viper with a total length of 628.2 mm (SVL 540.6 mm and TL 87.6 mm), preserved in 75% ethanol with its left hemipenes partially extruded (Figs 1, 2).

The head is slender and triangular shaped in dorsal view, distinct from the neck. Canthus rostralis are not distinct. The head is 25.2 mm in length, 13.2 mm in width and 8.2 mm in depth.

***Scalation*.** Rostral scale slightly up-turned, visible from dorsal view; nasal divided, anterior part larger; seven supralabials on both sides: second smallest, not reaching the pit; third highest, not touching the bottom of orbit (separated by one small subocular); fourth longest, not touching the orbit; three preoculars, two postoculars, inferior one touching the top of the third supralabials, forming the bottom margin of the orbit; two rows of temporals (2+4); infralabials 10 on left side while 11 on right, first pair in contact behind the mental; second, third and fourth pairs meet on the chin shield; chin shield is rhomboidal in shape, the posterior chin shield comprises two pairs of scales, forming the mental groove (Fig. 3A–C).

Dorsal scales in 23-21-15 rows (reducing from 19 to 18 posteriorly at ventral 94–96), keeled except for the first scale row bordering the ventral scales; ventral scales 165 (excluding four preventral scales); anal plate single; subcaudals 46, in pairs

***Coloration*.** Eye dark brown on the upper half while black on the bottom half, pupil black, vertical with light yellow margins; postorbital stripe wide, greyish brown and black bordered on the lower edge, extending from the posterior orbit to the ventral surface of the neck; supralabials and infralabials greyish brown, scattered with very small irregularly sized black blotches. One black triangular mark on the anterodorsal head, covering the caudomedial part of prefrontals. One bold black M-shaped mark on the dorsomedial head, covering the caudal part of lateral frontals, the lateral part of parietals, merged with the postorbital stripe at the largest temporal scale (but not covering the upper postorbital). The upper postorbital white while the top part of the bottom postorbital is black (covered by the postorbital stripe).

The body coloration is dark greyish brown, with two rows of irregular black annular crossbands on the mid-body, each covering 20 or more scales, separated by a gap of two row scale vertically, extending laterally to one or two dorsal scales from the ventrals. Ventral scales light grey, with two large black blotches on each side, clustered into two ventral stripes. The tip of tail is similar to the main body in coloration (Figs 1, 2).

***Skull*.** The description of the skull of *G.lipipengi* sp. nov. is based on the 3D-reconstructed model of the holotype.

***Snout*.** The premaxilla has bifurcated transverse process on each side. The anterior margin of the premaxilla is blunt. The dorsal tip of the ascending process of premaxilla is triangular in lateral view, not reaching the anterior tip of nasals. The horizontal laminae of the nasals are scutiform in dorsal view. The septomaxillae have prominent dorsolateral processes, nearly meeting the horizontal laminae of the nasals.

***Braincase*.** The parietal is roughly T-shaped in dorsal view. The anterolateral part of the parietal bulges prominently laterally while the dorsoposterior part tapers medially. The postorbital processes of the parietal are prominent. The frontals are squared. The lateral margin of frontals concaved obviously on each side, forming the dorsal edge of orbit. The prefrontal has an elongate blunt lateral process, posterolaterally pointed. The lacrimal foramen perforates the medial lamina of the prefrontal. The prefrontal-frontal join surface is waved in dorsal view.

The postorbital is relatively small and cashew-shaped, the top of the postorbital does not reach the posterolateral end of frontal. The basisphenoid is spearhead in shape, narrow anteriorly and expanded laterally. The supraoccipital is longitudinally compressed, occupies almost two thirds the total width of the otic region.

***Palatomaxillaryapparatus*.** The fang is relatively short and curved, roughly the same length of the maxilla, one third the length of ectopterygoid, attached with seven or eight replacement fangs on each side. The palatine bears three teeth. The ectopterygoid is flat and widened at the anterior part. The pterygoid is slender, the dentigerous process of pterygoid is straight, bearing 12/11 teeth (left/right), occupies almost half the total width of the pterygoid, the posterior portion of pterygoid is medially expanded.

***Suspensorium and mandible*.** The supratemporal is slender, has a lateral process, anterolaterally pointed, lies in front of the supratemporal-quadrate articulation. The quadrate is straight, slender, and enlarged on both ends. The mandible is slender and moderately curved. The prearticular crest of the compound bone is prominent while the surangular crest is slightly concaved. The dental bone bears 11/12 teeth (left/right); the dentary teeth are perpendicular to the dentary bone, decreasing in size at the third tooth. The posterior tip of ventral process of dentary extends farther posteriorly than the dorsal process.

***Dentition*.** Palatine: 3/3, pterygoid: 12/11, dentary: 11/12.

***Hemipenes*.** The hemipenes of *G.lipipengi* sp. nov. are generally similar to those of *G.rubromaculatus* and *G.huangi* but differ by the possession of longer and stronger spines, seven or eight subcaudals in length, and forked for two subcaudals. Small and stubby spines range from the basal to the distal side of the organ, without any conspicuously enlarged spines (versus 3–5 enlarged spines on the base in the *G.halys* complex; Gloyd 1990). The spines gradually increase in length distally.

###### Distribution and ecology.

**Figure 6.**
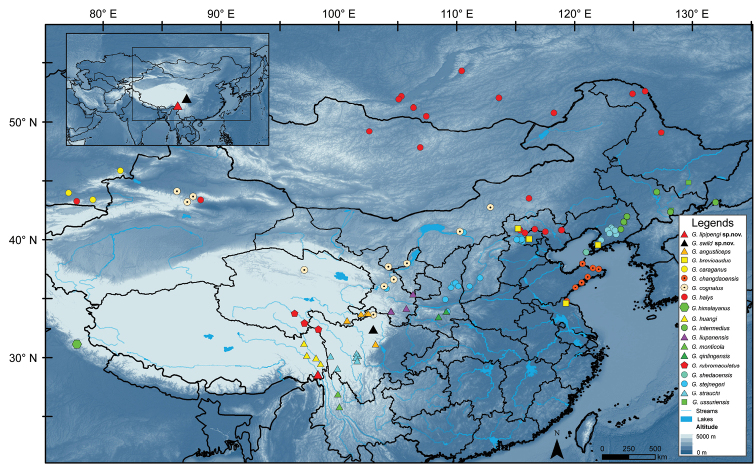
Type localities of *Gloydiuslipipengi* sp. nov. (red triangles) and *G.swild* sp. nov. (black triangles), with the collection localities of some other congeneric species.

**Figure 7.**
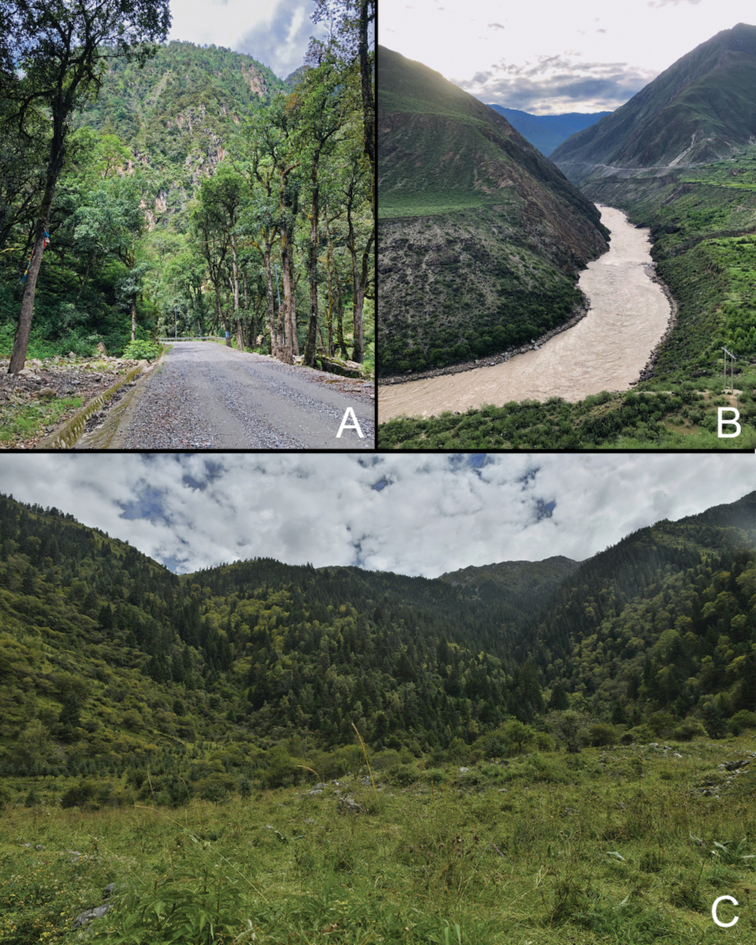
The habitat of *Gloydiuslipipengi* sp. nov. (**A** Muza Village, Zaty, Tibet, type locality of *G.lipipengi* sp. nov. **B** the landscape of the Nujiang River, 15 km from the type locality) and *Gloydiusswild* sp. nov. (**C** Heishui, Sichuan) **A** and **B** Photographs by Jin-Cheng Liu.

At present, *Gloydiuslipipengi* sp. nov. has only been reported from the type locality, Muza village, Zayu, Tibet, China (Fig. 6). The specimen was collected at 09:00 h on leaf litter in forest near the hot, dry valley on the lower reaches of the Nujiang River (Fig. 7). *Gloydiuslipipengi* sp. nov. accepted pink mice in captivity.

##### Gloydius swild

Taxon classificationAnimaliaSquamataViperidae

Shi & Malhotra sp. nov.

02CD0380-8A00-5BD8-A786-578E8E8E17E8

http://zoobank.org/77260121-7761-4D37-AC87-3FE77EEA378C

###### Etymology.

The new species from Heishui, Sichuan is named after the Swild Group (Southwest Wild, http://www.swild.cn/), who discovered the new species and collected the first species during an expedition to the Dagu Holy-glacier, Heishui, Sichuan. The common name of *G.swild* sp. nov. is suggested as “Glacier pit viper” in English, and “Bīng Chuān Fù (冰川蝮)” in Chinese.

###### Type series.

*Gloydiusswild* sp. nov, ***holotype***, IVPP OV2725 (G2, Figs 1, 3), adult female, collected from Heishui, Aba, Sichuan (32.23°N, 102.80°E, 2940 m), on 23 July, 2017, by the senior author; ***paratype***, IVPP OV 2726, adult female, the same locality as the holotype, collected by Jia-Wei Wu (chief executive officer of SWILD Group).

###### Diagnosis.

*Gloydiusswild* sp. nov. differs from other congeneric species in the following characteristics: i) the narrower postorbital stripe, ii) a pair of round spots on the parietal scales; iii) the absence of the black spots on the lateral body; iv) 21 rows of mid-body dorsal scales; v) a pair of arched stripes on the occiput; vi) 168–170 ventral scales, and vii) 43–46 subcaudal scales.

Morphologically, *Gloydiusswild* sp. nov. is quite similar to *G.angusticeps*, but differs by the narrower, straight bordered brown postorbital stripe (versus wider postorbital stripe with dentate lower border in *G.angusticeps*). *G.swild* sp. nov. differs from *G.strauchi*, *G.huangi*, and *G.rubromaculatus* by the narrow triangular head from dorsal view (versus spoon-shaped head in above-mentioned species), from *G.monticola* by having seven supralabials (versus always six supralabials) and more subcaudal scales (43–46 pairs versus always fewer than 30 pairs of subcaudal scales), from *G.qinlingensis* and *G.liupanensis* by its dark greyish brown background dorsal color (versus yellowish-brown body colour) and lacking a lateral white line on each side (versus possessing a lateral white line on each side), from *G.himalayanus* by possessing an indistinct canthus rostralis (versus very distinct canthus rostralis; Gloyd and Conant 1990).

###### Description of the holotype.

*Gloydiusswild* sp. nov., IVPP OV 2725, adult female, a slender pit viper with a total length of 529.5 mm (SVL 462 mm and TL 67.5 mm), preserved in 75% ethanol (Fig. 1).

The head is slender and narrow triangular shaped in dorsal view, distinct from the neck. Canthus rostralis not distinct. The head is 20.8 mm in length, 12.2 mm in width and 6.6 mm in depth.

***Scalation*.** Rostral scale slightly up-turned, visible from dorsal view; nasal divided, anterior part larger; seven supralabials on both sides: second smallest, not reaching the pit; third highest, not touching the bottom of orbit on the left (separated by one small subocular) while touching the bottom of the orbit on the right; fourth longest, not touching the orbit; three preoculars, two postoculars, inferior one touching the top of the third supralabials, forming the bottom margin of the orbit; two rows of temporals: 3+5/2+4 (L/R); infralabials 10, first pair in contact behind the mental; second, third and fourth pairs meet on the chin shield; chin shield is rhomboidal in shape, the posterior chin shield comprises two pairs of scales, forming the mental groove (Fig. 3D–F). Dorsal scales in 21-21-15 rows, keeled except for the first scale row bordering the ventral scales; ventral scales 170 (excluding four preventral scales); anal plate single; subcaudals 46, in pairs.

***Coloration*.***Gloydiusswild* sp. nov., eye light greyish brown on the upper half while black on the bottom half, pupil black, vertical with light yellow margins; postorbital stripe relatively narrow, only half the width of the anterior temporals, greyish brown and white bordered on the lower edge, extending from the posterior orbit to the lateral surface of the neck; supralabials and infralabials greyish white, scattered with large irregularly sized, black blotches, rendering the lateral head granitoid. One black Ω-shaped mark on the anterodorsal head, covering the posteromedial part of prefrontals, the anterior and lateral part of the frontals and the anterior part of the parietals. The infralabials are bordered with yellow on the lower edge.

The body coloration is dark blueish-grey, with two rows of irregular black X-shaped or C-shaped crossbands on the mid-body, each covering about 10 dorsal scales (or more), separated by a gap of one or two dorsal scales vertically, sometimes in contact with the adjacent ones forming zigzag stripes, but hardly merged on the medial dorsal line, extending laterally to one or two dorsal scales from the ventrals. Ventral scales light grey, scattered with dense irregular black blotches, rendering the ventral scales granitoid. The tip of tail is bony, similar to the main body in coloration on both ventral and dorsal sides (Figs 1C, D, 3D–F).

###### Infraspecific morphological variation.

Despite the inconspicuous variation in the coloration among the type series of *G.swild* sp. nov., the scalations vary considerably between the two specimens. The range of the temporal scales of the holotype (IVPP OV 2725) is 3+5 on the left side but 2+4 on the right side, while in the paratype (IVPP OV 2726), the temporal scales are displayed as 2+4 on both sides. Ventrals range from 168–170 in females (*n* = 2), while range from 43–46 in females (*n* = 3, including one shed skin from the wild). Total length ranges from 529.5–629.1 in adult females. The infralabials of the paratype lack the greyish-yellow margins seen in the holotype.

###### Distribution and ecology.

*Gloydiusswild* sp. nov. has been found in east part of Qinghai-Tibet plateau and Hengduanshan mountains, Heishui country, north Sichuan, about 15 km away from Dagu Holy-glacier National Geological Park, from along the route of Red Army’s long march (from June to August, 1935). They were found on or under the rocks (especially near the vegetations) on sunny slopes (Figs 6, 7C).

###### Viviparous reproduction.

One adult female (Holotype) was collected when pregnant, gave birth to eight neonates (including a couple of conjoined twins) on September 20^th^, 2017 in captivity. The weight of the normal neonates ranged between 3.00–3.45 g (3.01, 3.22, 3.22, 3.23, 3.28, 3.45, average = 3.235, *n* = 6). The weight of the conjoined twins was 2.86 g (weighed after the first shedding).

## Discussion

This study reveals the phylogenetic position of *G.himalayanus* within *Gloydius* for the first time. This study also reports two new *Gloydius* species, increasing the number of the recognized species in *Gloydius* to 23. The discovery of the new species has further verified the hypothesis that the Himalayan-Tibetan Plateau and Southwest Mountain Ranges should be considered as differentiation centres of Asian pit vipers. Furthermore, the discovery of *G.swild* sp. nov. suggests that the glaciers might be considered as key factors to the isolation and speciation of the alpine pit vipers in the southwest China.

Lastly, the systematic and taxonomic relationship of *G.qinlingensis* and *G.liupanensis* is still controversial. Despite their morphological similarities, these clades have not consistently formed a monophyletic group in earlier studies (Xu et al. 2012; Shi et al. 2017; Wang et al. 2019). In a subsequent study, which included more mitochondrial genes (specifically 16s rRNA) in the analysis (Li et al. 2020), *G.qinlingensis* and *G.liupanensis* formed a separate monophyletic lineage (Clade G) that is sister to the *G.halys* complex. The systematic position of the *G.qinlingensis*-*liupanensis* group that have been reconstructed in this study is consistent with Li et al. (2020).

In our analysis, the enigmatic clade formed by *Ovophisokinavensis* and *Trimeresurusgracilis* (Clade F) is basal to the genus *Gloydius*. As highlighted by Malhotra and Thorpe (2000), Tu et al. (2000), and numerous analyses since (Castoe and Parkinson 2006; Pyron et al. 2013; Alencar et al. 2016), the systematic status of these two species requires resolution.

As the squamation and body coloration variation is quite conservative within pit vipers (Gloyd and Conant 1990; Shi et al. 2017, 2018), it is necessary to obtain more specimens of the new species to investigate intraspecific variation in the new species. These data will be very helpful in verifying the stability of the diagnostic morphological characteristics of *G.lipipengi* sp. nov. Further fieldwork and molecular phylogenetics, particularly using nuclear genes, are still needed to investigate the origin, evolution, and migration of Asian pit vipers on the Qinghai-Tibet Plateau.

## Supplementary Material

XML Treatment for Gloydius lipipengi

XML Treatment for Gloydius swild
